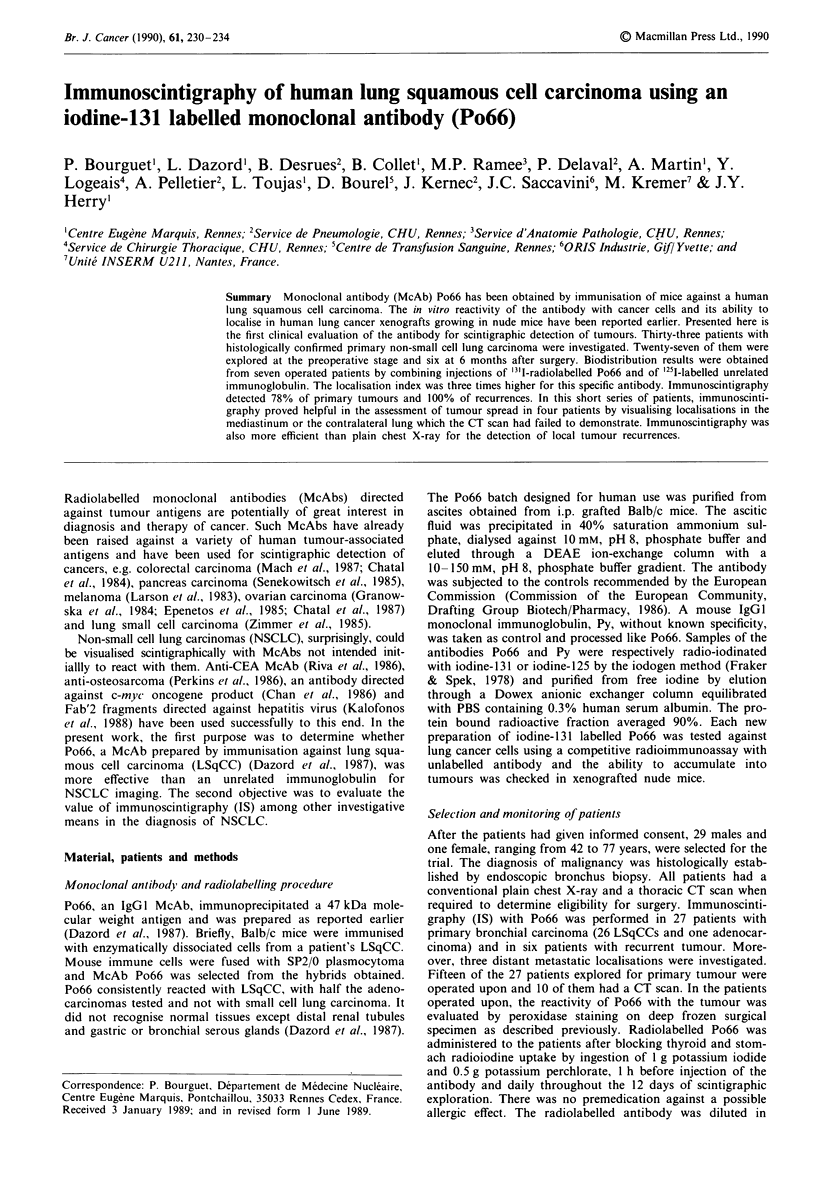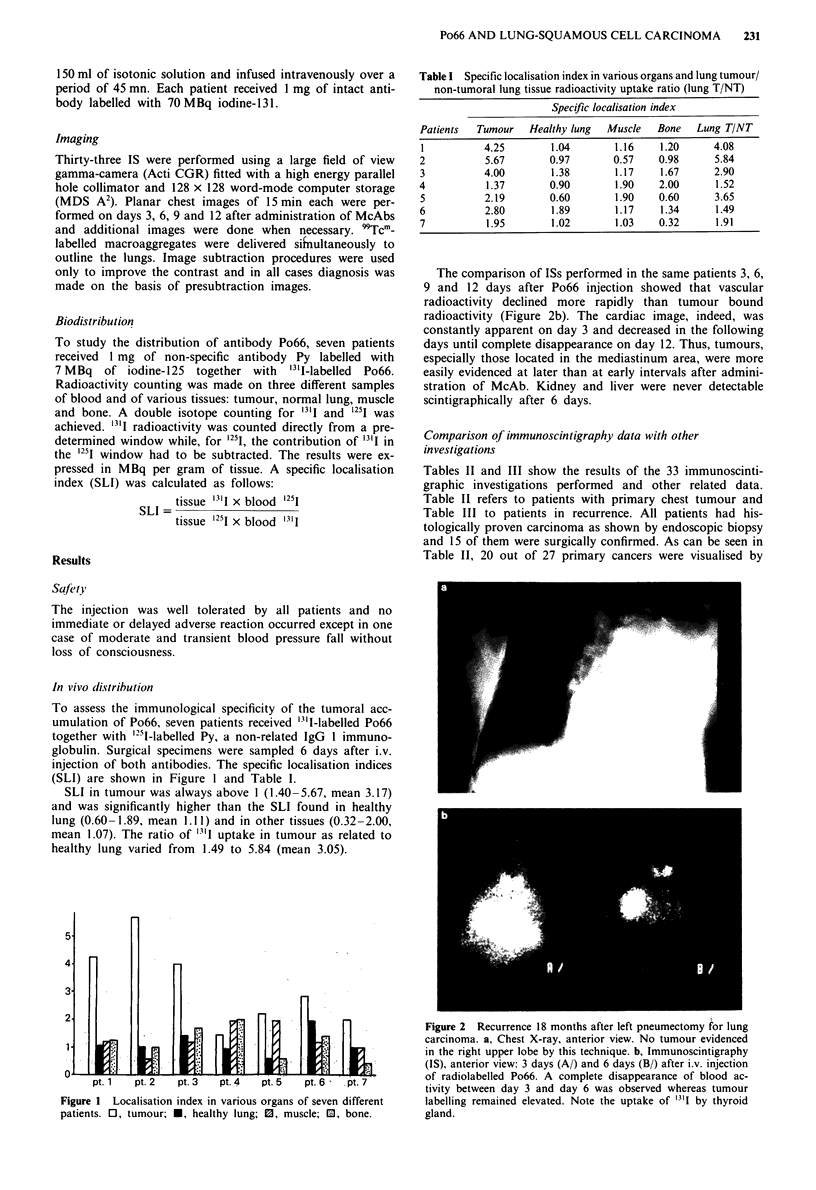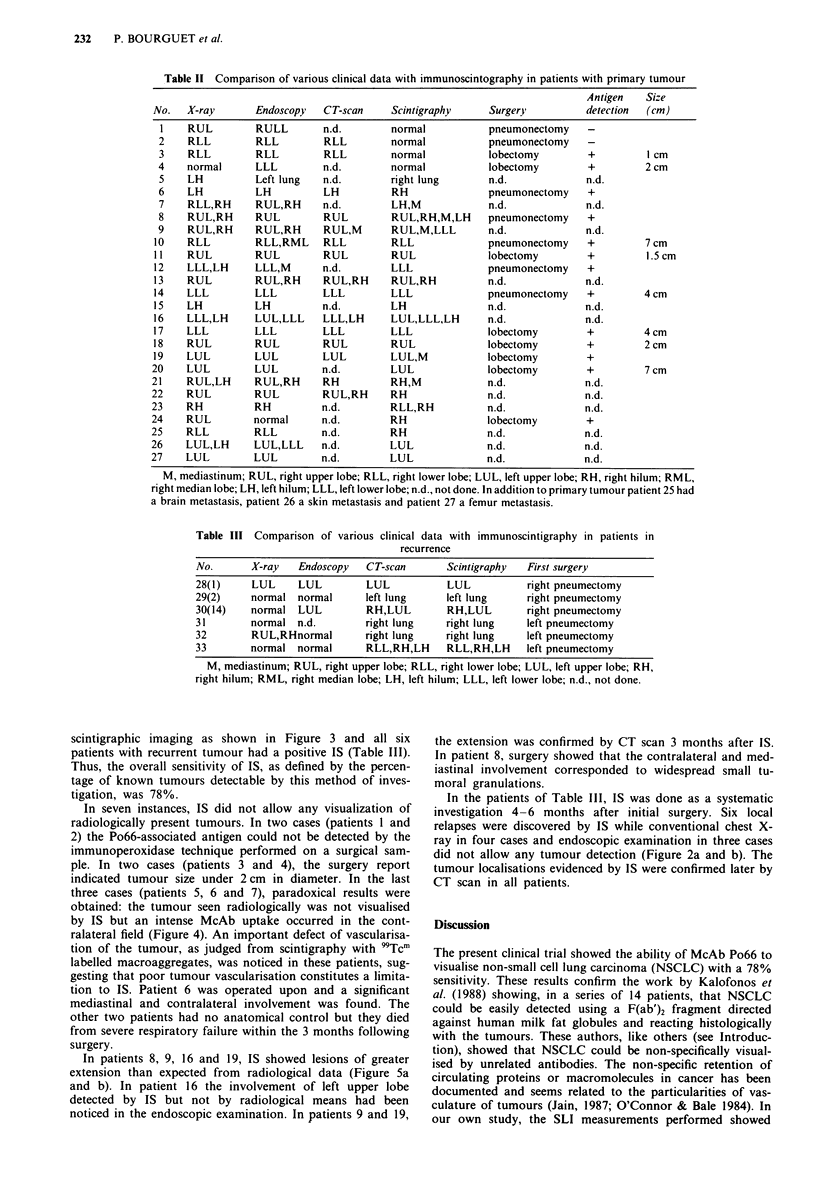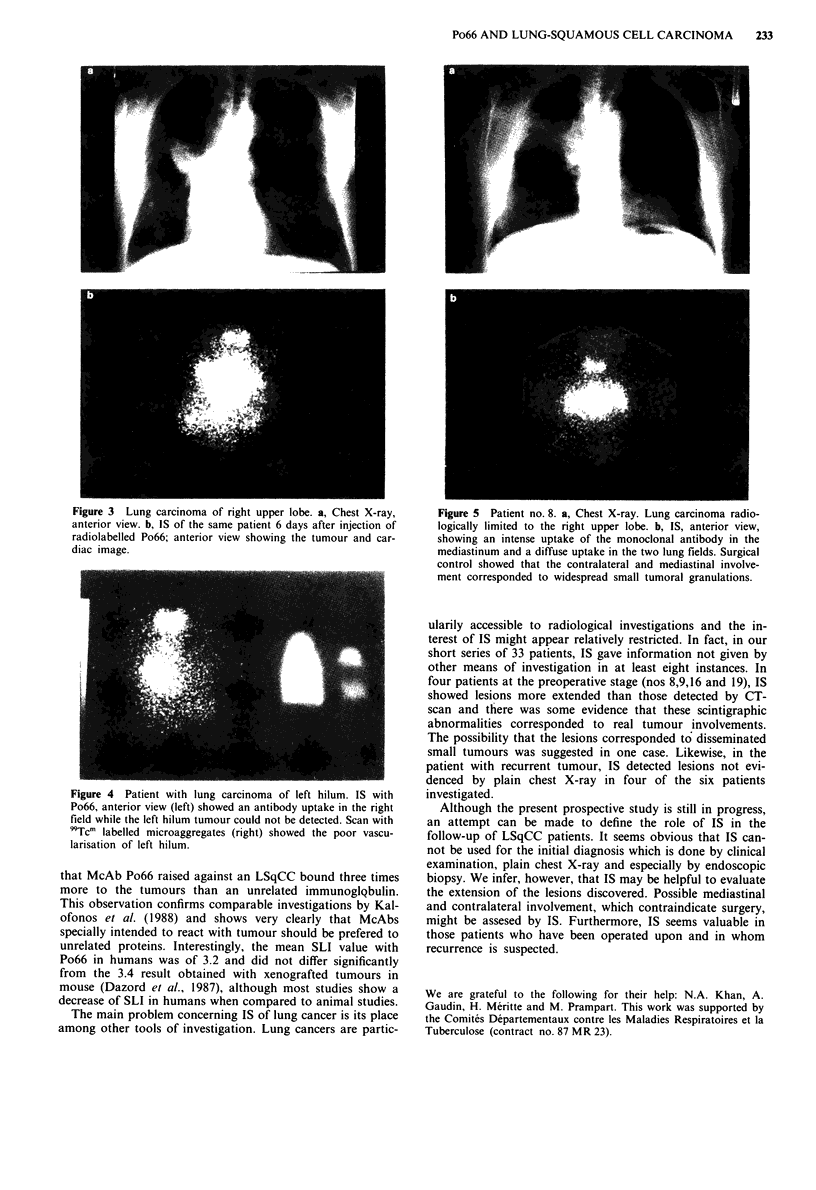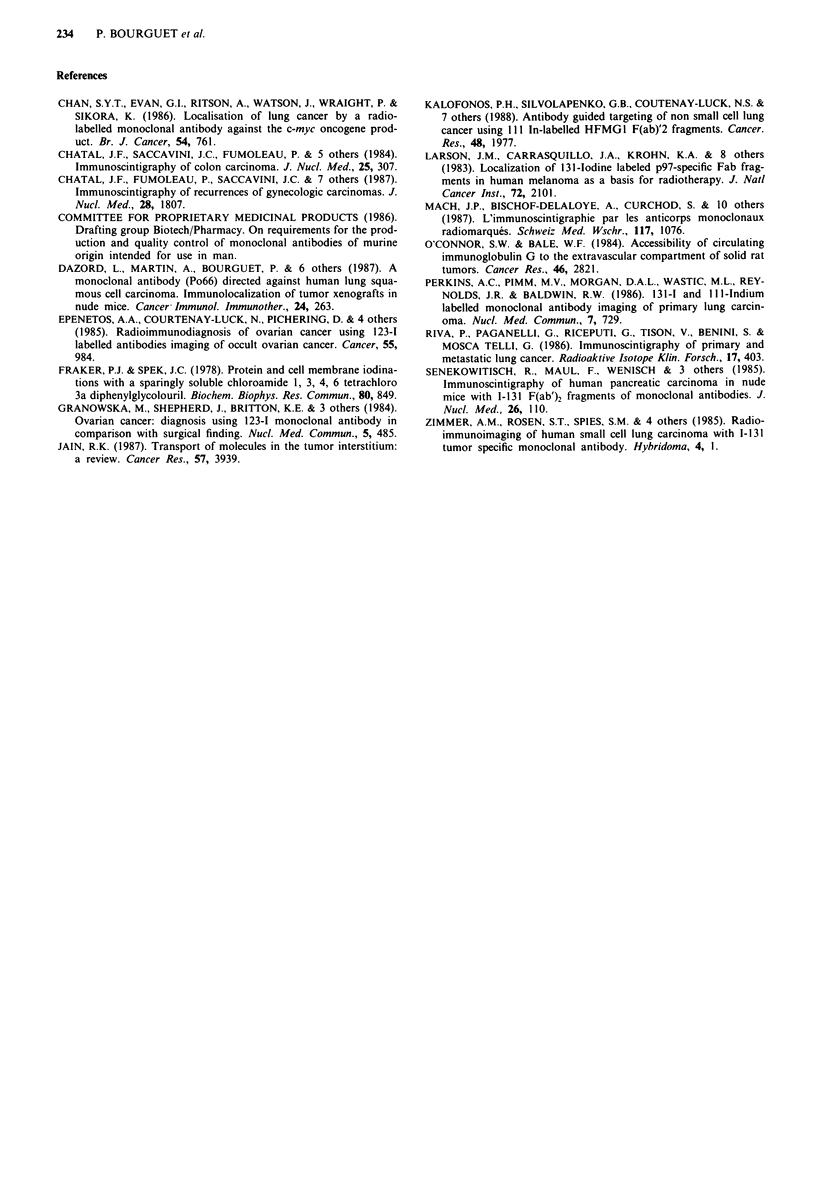# Immunoscintigraphy of human lung squamous cell carcinoma using an iodine-131 labelled monoclonal antibody (Po66).

**DOI:** 10.1038/bjc.1990.43

**Published:** 1990-02

**Authors:** P. Bourguet, L. Dazord, B. Desrues, B. Collet, M. P. Ramee, P. Delaval, A. Martin, Y. Logeais, A. Pelletier, L. Toujas

**Affiliations:** Centre Eugène Marquis, Rennes, France.

## Abstract

**Images:**


Br. J. Cancer (1990), 61, 230–234 © Macmillan Press Ltd., 1990

### Immunoscintigraphy of human lung squamous cell carcinoma using an iodine-131 labelled monoclonal antibody (Po66)

P. Bourguet[1], L. Dazord[1], B. Desrues[2], B. Collet[1], M.P. Ramee[3], P. Delaval[2], A. Martin[1], Y. Logeais[4], A. Pelletier[2], L. Toujas[1], D. Bourel[5], J. Kernec[2], J.C. Saccavini[6], M. Kremer[7] & J.Y. Herry[1]

[1]*Centre Eugène Marquis, Rennes;* [2]*Service de Pneumologie, CHU, Rennes;* [3]*Service d'Anatomie Pathologie, CHU, Rennes;* [4]*Service de Chirurgie Thoracique, CHU, Rennes;* [5]*Centre de Transfusion Sanguine, Rennes;* [6]*ORIS Industrie, Gif/Yvette; and* [7]*Unité INSERM U211, Nantes, France.*

**Summary** Monoclonal antibody (McAb) Po66 has been obtained by immunisation of mice against a human lung squamous cell carcinoma. The *in vitro* reactivity of the antibody with cancer cells and its ability to localise in human lung cancer xenografts growing in nude mice have been reported earlier. Presented here is the first clinical evaluation of the antibody for scintigraphic detection of tumours. Thirty-three patients with histologically confirmed primary non-small cell lung carcinoma were investigated. Twenty-seven of them were explored at the preoperative stage and six at 6 months after surgery. Biodistribution results were obtained from seven operated patients by combining injections of $^{131}$I-radiolabelled Po66 and of $^{125}$I-labelled unrelated immunoglobulin. The localisation index was three times higher for this specific antibody. Immunoscintigraphy detected 78% of primary tumours and 100% of recurrences. In this short series of patients, immunoscintigraphy proved helpful in the assessment of tumour spread in four patients by visualising localisations in the mediastinum or the contralateral lung which the CT scan had failed to demonstrate. Immunoscintigraphy was also more efficient than plain chest X-ray for the detection of local tumour recurrences.

Radiolabelled monoclonal antibodies (McAbs) directed against tumour antigens are potentially of great interest in diagnosis and therapy of cancer. Such McAbs have already been raised against a variety of human tumour-associated antigens and have been used for scintigraphic detection of cancers, e.g. colorectal carcinoma (Mach *et al.*, 1987; Chatal *et al.*, 1984), pancreas carcinoma (Senekowitsch *et al.*, 1985), melanoma (Larson *et al.*, 1983), ovarian carcinoma (Granowska *et al.*, 1984; Epenetos *et al.*, 1985; Chatal *et al.*, 1987) and lung small cell carcinoma (Zimmer *et al.*, 1985).

Non-small cell lung carcinomas (NSCLC), surprisingly, could be visualised scintigraphically with McAbs not intended initiallly to react with them. Anti-CEA McAb (Riva *et al.*, 1986), anti-osteosarcoma (Perkins *et al.*, 1986), an antibody directed against c-*myc* oncogene product (Chan *et al.*, 1986) and Fab'2 fragments directed against hepatitis virus (Kalofonos *et al.*, 1988) have been used successfully to this end. In the present work, the first purpose was to determine whether Po66, a McAb prepared by immunisation against lung squamous cell carcinoma (LSqCC) (Dazord *et al.*, 1987), was more effective than an unrelated immunoglobulin for NSCLC imaging. The second objective was to evaluate the value of immunoscintigraphy (IS) among other investigative means in the diagnosis of NSCLC.

### Material, patients and methods

### *Monoclonal antibody and radiolabelling procedure*

Po66, an IgG1 McAb, immunoprecipitated a 47 kDa molecular weight antigen and was prepared as reported earlier (Dazord *et al.*, 1987). Briefly, Balb/c mice were immunised with enzymatically dissociated cells from a patient's LSqCC. Mouse immune cells were fused with SP2/0 plasmocytoma and McAb Po66 was selected from the hybrids obtained. Po66 consistently reacted with LSqCC, with half the adenocarcinomas tested and not with small cell lung carcinoma. It did not recognise normal tissues except distal renal tubules and gastric or bronchial serous glands (Dazord *et al.*, 1987).

The Po66 batch designed for human use was purified from ascites obtained from i.p. grafted Balb/c mice. The ascitic fluid was precipitated in 40% saturation ammonium sulphate, dialysed against 10 mM, pH 8, phosphate buffer and eluted through a DEAE ion-exchange column with a 10–150 mM, pH 8, phosphate buffer gradient. The antibody was subjected to the controls recommended by the European Commission (Commission of the European Community, Drafting Group Biotech/Pharmacy, 1986). A mouse IgG1 monoclonal immunoglobulin, Py, without known specificity, was taken as control and processed like Po66. Samples of the antibodies Po66 and Py were respectively radio-iodinated with iodine-131 or iodine-125 by the iodogen method (Fraker & Spek, 1978) and purified from free iodine by elution through a Dowex anionic exchanger column equilibrated with PBS containing 0.3% human serum albumin. The protein bound radioactive fraction averaged 90%. Each new preparation of iodine-131 labelled Po66 was tested against lung cancer cells using a competitive radioimmunoassay with unlabelled antibody and the ability to accumulate into tumours was checked in xenografted nude mice.

### *Selection and monitoring of patients*

After the patients had given informed consent, 29 males and one female, ranging from 42 to 77 years, were selected for the trial. The diagnosis of malignancy was histologically established by endoscopic bronchus biopsy. All patients had a conventional plain chest X-ray and a thoracic CT scan when required to determine eligibility for surgery. Immunoscintigraphy (IS) with Po66 was performed in 27 patients with primary bronchial carcinoma (26 LSqCCs and one adenocarcinoma) and in six patients with recurrent tumour. Moreover, three distant metastatic localisations were investigated. Fifteen of the 27 patients explored for primary tumour were operated upon and 10 of them had a CT scan. In the patients operated upon, the reactivity of Po66 with the tumour was evaluated by peroxidase staining on deep frozen surgical specimen as described previously. Radiolabelled Po66 was administered to the patients after blocking thyroid and stomach radioiodine uptake by ingestion of 1 g potassium iodide and 0.5 g potassium perchlorate, 1 h before injection of the antibody and daily throughout the 12 days of scintigraphic exploration. There was no premedication against a possible allergic effect. The radiolabelled antibody was diluted in

Correspondence: P. Bourguet, Département de Médecine Nucléaire, Centre Eugène Marquis, Pontchaillou, 35033 Rennes Cedex, France.
Received 3 January 1989; and in revised form 1 June 1989.

150 ml of isotonic solution and infused intravenously over a period of 45 mn. Each patient received 1 mg of intact antibody labelled with 70 MBq iodine-131.

### *Imaging*

Thirty-three IS were performed using a large field of view gamma-camera (Acti CGR) fitted with a high energy parallel hole collimator and 128 × 128 word-mode computer storage (MDS $A^2$). Planar chest images of 15 min each were performed on days 3, 6, 9 and 12 after administration of McAbs and additional images were done when necessary. $^{99}Tc^m$-labelled macroaggregates were delivered simultaneously to outline the lungs. Image subtraction procedures were used only to improve the contrast and in all cases diagnosis was made on the basis of presubtraction images.

### *Biodistribution*

To study the distribution of antibody Po66, seven patients received 1 mg of non-specific antibody Py labelled with 7 MBq of iodine-125 together with $^{131}$I-labelled Po66. Radioactivity counting was made on three different samples of blood and of various tissues: tumour, normal lung, muscle and bone. A double isotope counting for $^{131}$I and $^{125}$I was achieved. $^{131}$I radioactivity was counted directly from a predetermined window while, for $^{125}$I, the contribution of $^{131}$I in the $^{125}$I window had to be subtracted. The results were expressed in MBq per gram of tissue. A specific localisation index (SLI) was calculated as follows:

$$SLI = \frac{\text{tissue } ^{131}I \times \text{blood } ^{125}I}{\text{tissue } ^{125}I \times \text{blood } ^{131}I}$$

### Results

### *Safety*

The injection was well tolerated by all patients and no immediate or delayed adverse reaction occurred except in one case of moderate and transient blood pressure fall without loss of consciousness.

### *In vivo distribution*

To assess the immunological specificity of the tumoral accumulation of Po66, seven patients received $^{131}$I-labelled Po66 together with $^{125}$I-labelled Py, a non-related IgG 1 immunoglobulin. Surgical specimens were sampled 6 days after i.v. injection of both antibodies. The specific localisation indices (SLI) are shown in Figure 1 and Table I.

SLI in tumour was always above 1 (1.40–5.67, mean 3.17) and was significantly higher than the SLI found in healthy lung (0.60–1.89, mean 1.11) and in other tissues (0.32–2.00, mean 1.07). The ratio of $^{131}$I uptake in tumour as related to healthy lung varied from 1.49 to 5.84 (mean 3.05).

**Figure 1** Localisation index in various organs of seven different patients. □, tumour; ■, healthy lung; ▨, muscle; ▩, bone.

**Table I** Specific localisation index in various organs and lung tumour/non-tumoral lung tissue radioactivity uptake ratio (lung T/NT)

| | *Specific localisation index* | | | | |
|---|---|---|---|---|---|
| *Patients* | *Tumour* | *Healthy lung* | *Muscle* | *Bone* | *Lung T/NT* |
| 1 | 4.25 | 1.04 | 1.16 | 1.20 | 4.08 |
| 2 | 5.67 | 0.97 | 0.57 | 0.98 | 5.84 |
| 3 | 4.00 | 1.38 | 1.17 | 1.67 | 2.90 |
| 4 | 1.37 | 0.90 | 1.90 | 2.00 | 1.52 |
| 5 | 2.19 | 0.60 | 1.90 | 0.60 | 3.65 |
| 6 | 2.80 | 1.89 | 1.17 | 1.34 | 1.49 |
| 7 | 1.95 | 1.02 | 1.03 | 0.32 | 1.91 |

The comparison of ISs performed in the same patients 3, 6, 9 and 12 days after Po66 injection showed that vascular radioactivity declined more rapidly than tumour bound radioactivity (Figure 2b). The cardiac image, indeed, was constantly apparent on day 3 and decreased in the following days until complete disappearance on day 12. Thus, tumours, especially those located in the mediastinum area, were more easily evidenced at later than at early intervals after administration of McAb. Kidney and liver were never detectable scintigraphically after 6 days.

### *Comparison of immunoscintigraphy data with other investigations*

Tables II and III show the results of the 33 immunoscintigraphic investigations performed and other related data. Table II refers to patients with primary chest tumour and Table III to patients in recurrence. All patients had histologically proven carcinoma as shown by endoscopic biopsy and 15 of them were surgically confirmed. As can be seen in Table II, 20 out of 27 primary cancers were visualised by

**Figure 2** Recurrence 18 months after left pneumectomy for lung carcinoma. **a**, Chest X-ray, anterior view. No tumour evidenced in the right upper lobe by this technique. **b**, Immunoscintigraphy (IS), anterior view: 3 days (A/) and 6 days (B/) after i.v. injection of radiolabelled Po66. A complete disappearance of blood activity between day 3 and day 6 was observed whereas tumour labelling remained elevated. Note the uptake of $^{131}$I by thyroid gland.

**Table II** Comparison of various clinical data with immunoscintography in patients with primary tumour

| No. | X-ray | Endoscopy | CT-scan | Scintigraphy | Surgery | Antigen detection | Size (cm) |
|---|---|---|---|---|---|---|---|
| 1 | RUL | RULL | n.d. | normal | pneumonectomy | – | |
| 2 | RLL | RLL | RLL | normal | pneumonectomy | – | |
| 3 | RLL | RLL | RLL | normal | lobectomy | + | 1 cm |
| 4 | normal | LLL | n.d. | normal | lobectomy | + | 2 cm |
| 5 | LH | Left lung | n.d. | right lung | n.d. | n.d. | |
| 6 | LH | LH | LH | RH | pneumonectomy | + | |
| 7 | RLL,RH | RUL,RH | n.d. | LH,M | n.d. | n.d. | |
| 8 | RUL,RH | RUL | RUL | RUL,RH,M,LH | pneumonectomy | + | |
| 9 | RUL,RH | RUL,RH | RUL,M | RUL,M,LLL | n.d. | n.d. | |
| 10 | RLL | RLL,RML | RLL | RLL | pneumonectomy | + | 7 cm |
| 11 | RUL | RUL | RUL | RUL | lobectomy | + | 1.5 cm |
| 12 | LLL,LH | LLL,M | n.d. | LLL | pneumonectomy | + | |
| 13 | RUL | RUL,RH | RUL,RH | RUL,RH | n.d. | n.d. | |
| 14 | LLL | LLL | LLL | LLL | pneumonectomy | + | 4 cm |
| 15 | LH | LH | n.d. | LH | n.d. | n.d. | |
| 16 | LLL,LH | LUL,LLL | LLL,LH | LUL,LLL,LH | n.d. | n.d. | |
| 17 | LLL | LLL | LLL | LLL | lobectomy | + | 4 cm |
| 18 | RUL | RUL | RUL | RUL | lobectomy | + | 2 cm |
| 19 | LUL | LUL | LUL | LUL,M | lobectomy | + | |
| 20 | LUL | LUL | n.d. | LUL | lobectomy | + | 7 cm |
| 21 | RUL,LH | RUL,RH | RH | RH,M | n.d. | n.d. | |
| 22 | RUL | RUL | RUL,RH | RH | n.d. | n.d. | |
| 23 | RH | RH | n.d. | RLL,RH | n.d. | n.d. | |
| 24 | RUL | normal | n.d. | RH | lobectomy | + | |
| 25 | RLL | RLL | n.d. | RH | n.d. | n.d. | |
| 26 | LUL,LH | LUL,LLL | n.d. | LUL | n.d. | n.d. | |
| 27 | LUL | LUL | n.d. | LUL | n.d. | n.d. | |

M, mediastinum; RUL, right upper lobe; RLL, right lower lobe; LUL, left upper lobe; RH, right hilum; RML, right median lobe; LH, left hilum; LLL, left lower lobe; n.d., not done. In addition to primary tumour patient 25 had a brain metastasis, patient 26 a skin metastasis and patient 27 a femur metastasis.

**Table III** Comparison of various clinical data with immunoscintigraphy in patients in recurrence

| No. | X-ray | Endoscopy | CT-scan | Scintigraphy | First surgery |
|---|---|---|---|---|---|
| 28(1) | LUL | LUL | LUL | LUL | right pneumectomy |
| 29(2) | normal | normal | left lung | left lung | right pneumectomy |
| 30(14) | normal | LUL | RH,LUL | RH,LUL | right pneumectomy |
| 31 | normal | n.d. | right lung | right lung | left pneumectomy |
| 32 | RUL,RH | normal | right lung | right lung | left pneumectomy |
| 33 | normal | normal | RLL,RH,LH | RLL,RH,LH | left pneumectomy |

M, mediastinum; RUL, right upper lobe; RLL, right lower lobe; LUL, left upper lobe; RH, right hilum; RML, right median lobe; LH, left hilum; LLL, left lower lobe; n.d., not done.

scintigraphic imaging as shown in Figure 3 and all six patients with recurrent tumour had a positive IS (Table III). Thus, the overall sensitivity of IS, as defined by the percentage of known tumours detectable by this method of investigation, was 78%.

In seven instances, IS did not allow any visualization of radiologically present tumours. In two cases (patients 1 and 2) the Po66-associated antigen could not be detected by the immunoperoxidase technique performed on a surgical sample. In two cases (patients 3 and 4), the surgery report indicated tumour size under 2 cm in diameter. In the last three cases (patients 5, 6 and 7), paradoxical results were obtained: the tumour seen radiologically was not visualised by IS but an intense McAb uptake occurred in the contralateral field (Figure 4). An important defect of vascularisation of the tumour, as judged from scintigraphy with $^{99}Tc^{m}$ labelled macroaggregates, was noticed in these patients, suggesting that poor tumour vascularisation constitutes a limitation to IS. Patient 6 was operated upon and a significant mediastinal and contralateral involvement was found. The other two patients had no anatomical control but they died from severe respiratory failure within the 3 months following surgery.

In patients 8, 9, 16 and 19, IS showed lesions of greater extension than expected from radiological data (Figure 5a and b). In patient 16 the involvement of left upper lobe detected by IS but not by radiological means had been noticed in the endoscopic examination. In patients 9 and 19, the extension was confirmed by CT scan 3 months after IS. In patient 8, surgery showed that the contralateral and mediastinal involvement corresponded to widespread small tumoral granulations.

In the patients of Table III, IS was done as a systematic investigation 4–6 months after initial surgery. Six local relapses were discovered by IS while conventional chest X-ray in four cases and endoscopic examination in three cases did not allow any tumour detection (Figure 2a and b). The tumour localisations evidenced by IS were confirmed later by CT scan in all patients.

### Discussion

The present clinical trial showed the ability of McAb Po66 to visualise non-small cell lung carcinoma (NSCLC) with a 78% sensitivity. These results confirm the work by Kalofonos *et al.* (1988) showing, in a series of 14 patients, that NSCLC could be easily detected using a $F(ab')_2$ fragment directed against human milk fat globules and reacting histologically with the tumours. These authors, like others (see Introduction), showed that NSCLC could be non-specifically visualised by unrelated antibodies. The non-specific retention of circulating proteins or macromolecules in cancer has been documented and seems related to the particularities of vasculature of tumours (Jain, 1987; O'Connor & Bale 1984). In our own study, the SLI measurements performed showed

**Figure 3** Lung carcinoma of right upper lobe. **a**, Chest X-ray, anterior view. **b**, IS of the same patient 6 days after injection of radiolabelled Po66; anterior view showing the tumour and cardiac image.

**Figure 4** Patient with lung carcinoma of left hilum. IS with Po66, anterior view (left) showed an antibody uptake in the right field while the left hilum tumour could not be detected. Scan with $^{99}Tc^{m}$ labelled microaggregates (right) showed the poor vascularisation of left hilum.

that McAb Po66 raised against an LSqCC bound three times more to the tumours than an unrelated immunoglobulin. This observation confirms comparable investigations by Kalofonos *et al.* (1988) and shows very clearly that McAbs specially intended to react with tumour should be prefered to unrelated proteins. Interestingly, the mean SLI value with Po66 in humans was of 3.2 and did not differ significantly from the 3.4 result obtained with xenografted tumours in mouse (Dazord *et al.*, 1987), although most studies show a decrease of SLI in humans when compared to animal studies.

The main problem concerning IS of lung cancer is its place among other tools of investigation. Lung cancers are partic-

**Figure 5** Patient no. 8. **a**, Chest X-ray. Lung carcinoma radiologically limited to the right upper lobe. **b**, IS, anterior view, showing an intense uptake of the monoclonal antibody in the mediastinum and a diffuse uptake in the two lung fields. Surgical control showed that the contralateral and mediastinal involvement corresponded to widespread small tumoral granulations.

ularly accessible to radiological investigations and the interest of IS might appear relatively restricted. In fact, in our short series of 33 patients, IS gave information not given by other means of investigation in at least eight instances. In four patients at the preoperative stage (nos 8,9,16 and 19), IS showed lesions more extended than those detected by CT-scan and there was some evidence that these scintigraphic abnormalities corresponded to real tumour involvements. The possibility that the lesions corresponded to disseminated small tumours was suggested in one case. Likewise, in the patient with recurrent tumour, IS detected lesions not evidenced by plain chest X-ray in four of the six patients investigated.

Although the present prospective study is still in progress, an attempt can be made to define the role of IS in the follow-up of LSqCC patients. It seems obvious that IS cannot be used for the initial diagnosis which is done by clinical examination, plain chest X-ray and especially by endoscopic biopsy. We infer, however, that IS may be helpful to evaluate the extension of the lesions discovered. Possible mediastinal and contralateral involvement, which contraindicate surgery, might be assesed by IS. Furthermore, IS seems valuable in those patients who have been operated upon and in whom recurrence is suspected.

We are grateful to the following for their help: N.A. Khan, A. Gaudin, H. Méritte and M. Prampart. This work was supported by the Comités Départementaux contre les Maladies Respiratoires et la Tuberculose (contract no. 87 MR 23).

### References

CHAN, S.Y.T., EVAN, G.I., RITSON, A., WATSON, J., WRAIGHT, P. & SIKORA, K. (1986). Localisation of lung cancer by a radiolabelled monoclonal antibody against the c-*myc* oncogene product. *Br. J. Cancer*, **54**, 761.

CHATAL, J.F., SACCAVINI, J.C., FUMOLEAU, P. & 5 others (1984). Immunoscintigraphy of colon carcinoma. *J. Nucl. Med.*, **25**, 307.

CHATAL, J.F., FUMOLEAU, P., SACCAVINI, J.C. & 7 others (1987). Immunoscintigraphy of recurrences of gynecologic carcinomas. *J. Nucl. Med.*, **28**, 1807.

COMMITTEE FOR PROPRIETARY MEDICINAL PRODUCTS (1986). Drafting group Biotech/Pharmacy. On requirements for the production and quality control of monoclonal antibodies of murine origin intended for use in man.

DAZORD, L., MARTIN, A., BOURGUET, P. & 6 others (1987). A monoclonal antibody (Po66) directed against human lung squamous cell carcinoma. Immunolocalization of tumor xenografts in nude mice. *Cancer Immunol. Immunother.*, **24**, 263.

EPENETOS, A.A., COURTENAY-LUCK, N., PICHERING, D. & 4 others (1985). Radioimmunodiagnosis of ovarian cancer using 123-I labelled antibodies imaging of occult ovarian cancer. *Cancer*, **55**, 984.

FRAKER, P.J. & SPEK, J.C. (1978). Protein and cell membrane iodinations with a sparingly soluble chloroamide 1, 3, 4, 6 tetrachloro 3a diphenylglycolouril. *Biochem. Biophys. Res. Commun.*, **80**, 849.

GRANOWSKA, M., SHEPHERD, J., BRITTON, K.E. & 3 others (1984). Ovarian cancer: diagnosis using 123-I monoclonal antibody in comparison with surgical finding. *Nucl. Med. Commun.*, **5**, 485.

JAIN, R.K. (1987). Transport of molecules in the tumor interstitium: a review. *Cancer Res.*, **57**, 3939.

KALOFONOS, P.H., SILVOLAPENKO, G.B., COUTENAY-LUCK, N.S. & 7 others (1988). Antibody guided targeting of non small cell lung cancer using 111 In-labelled HFMG1 F(ab)'2 fragments. *Cancer. Res.*, **48**, 1977.

LARSON, J.M., CARRASQUILLO, J.A., KROHN, K.A. & 8 others (1983). Localization of 131-Iodine labeled p97-specific Fab fragments in human melanoma as a basis for radiotherapy. *J. Natl Cancer Inst.*, **72**, 2101.

MACH, J.P., BISCHOF-DELALOYE, A., CURCHOD, S. & 10 others (1987). L'immunoscintigraphie par les anticorps monoclonaux radiomarqués. *Schweiz Med. Wschr.*, **117**, 1076.

O'CONNOR, S.W. & BALE, W.F. (1984). Accessibility of circulating immunoglobulin G to the extravascular compartment of solid rat tumors. *Cancer Res.*, **46**, 2821.

PERKINS, A.C., PIMM, M.V., MORGAN, D.A.L., WASTIC, M.L., REYNOLDS, J.R. & BALDWIN, R.W. (1986). 131-I and 111-Indium labelled monoclonal antibody imaging of primary lung carcinoma. *Nucl. Med. Commun.*, **7**, 729.

RIVA, P., PAGANELLI, G., RICEPUTI, G., TISON, V., BENINI, S. & MOSCA TELLI, G. (1986). Immunoscintigraphy of primary and metastatic lung cancer. *Radioaktive Isotope Klin. Forsch.*, **17**, 403.

SENEKOWITISCH, R., MAUL, F., WENISCH & 3 others (1985). Immunoscintigraphy of human pancreatic carcinoma in nude mice with I-131 $F(ab')_2$ fragments of monoclonal antibodies. *J. Nucl. Med.*, **26**, 110.

ZIMMER, A.M., ROSEN, S.T., SPIES, S.M. & 4 others (1985). Radioimmunoimaging of human small cell lung carcinoma with I-131 tumor specific monoclonal antibody. *Hybridoma*, **4**, 1.